# Historical biogeography and ecological niche modelling of the *Asimina-Disepalum* clade (Annonaceae): role of ecological differentiation in Neotropical-Asian disjunctions and diversification in Asia

**DOI:** 10.1186/s12862-017-1038-4

**Published:** 2017-08-14

**Authors:** Pui-Sze Li, Daniel C. Thomas, Richard M. K. Saunders

**Affiliations:** 1 School of Biological Sciences, The University of Hong Kong, Pokfulam Road, Hong Kong, China; 2 Current address: Singapore Botanic Gardens, 1 Cluny Road, Singapore, 259569 Singapore

**Keywords:** Boreotropical forests, Neotropical-Asian disjunction, Molecular dating, Ancestral area optimization, Ecological niche modelling, *Asimina*, *Disepalum*, Annonaceae

## Abstract

**Background:**

The *Asimina-Disepalum* clade (Annonaceae subfam. Annonoideae tribe Annoneae) includes a major Neotropical-Asian biogeographical disjunction. We evaluate whether this disjunction can be explained by the Eocene boreotropics hypothesis, which relies on the existence of extensive boreotropical forests during the Late Palaeocene-Early Eocene thermal maximum (52–50 Ma), followed by disruption of boreotropical vegetation during post-Eocene cooling. Molecular dating using an uncorrelated relaxed molecular clock (UCLD) model with two fossil calibrations, ancestral range estimation, and ecological niche modelling across evolutionary time were performed. Our focus was the geographical origin of *Disepalum* and general biogeographic patterns within this genus. Comparison of ecological tolerance among extant species and niche reconstructions at ancestral nodes within the clade enabled insights in likely migration routes of lineages, as well as evaluating the role of bioclimatic ecological differentiation in the diversification of *Disepalum* within Southeast Asia.

**Results:**

The inferred vicariance event associated with the *Asimina*-*Disepalum* disjunction is estimated to have originated ca. 40 Mya [95% highest posterior density (HPD): 44.3–35.5 Mya]. The *Disepalum* crown lineage is estimated to have originated ca. 9 Mya (95% HPD: 10.6–7.6), either in western Malesia and continental Southeast Asia, or exclusively in western Malesia. Ecological niche modelling shows that seasonality of temperature and precipitation are major contributors determining the geographical range of species. Ancestral niche modelling furthermore indicates that the ancestor of the *Asimina-Disepalum* clade likely had bioclimatic preferences close to conditions found in current tropical and subtropical climates across Asia, whereas the ancestors of the *Asimina* and *Disepalum* crown groups are projected onto the more subtropical and tropical regions, respectively.

**Conclusions:**

The vicariance event associated with the Neotropical-Asian disjunction within the *Asimina-Disepalum* clade likely coincided with climatic deterioration at the Eocene-Oligocene boundary. Although detrended component analyses (DCA) indicate that altitude and seasonality of temperature and precipitation have the greatest influence in determining the geographical range of species, isolation due to palaeogeographic and palaeoclimatic events appears to be of greater significance than climate niche differentiation in driving diversification in *Disepalum*.

**Electronic supplementary material:**

The online version of this article (doi:10.1186/s12862-017-1038-4) contains supplementary material, which is available to authorized users.

## Background

The species-rich early divergent angiosperm family Annonaceae (ca. 2300 species: [1]) is considered to be ecologically highly conservative [2, 3], with most genera restricted to wet or seasonally dry tropical lowland forests. The family has been used as a study system to investigate tropical forest biogeography and evolution because of its wide distribution, species richness and ecological importance in Palaeotropical and Neotropical forests, as well its presumed high degree of climate niche conservatism during evolution (e.g. [3, 4]).

Intercontinental tropical disjunctions—in which species in sister clades are geographically restricted to tropical regions in different continents—are important biogeographical patterns that have been identified in numerous flowering plant taxa (e.g. [5, 6]). Within the Annonaceae, numerous examples have been identified, including at least nine disjunctions between the western Palaeotropics (African-Madagascar) and the eastern Palaeotropics (Asia, Australia) [2, 4, 7–9], three disjunctions between Africa and the Neotropics [2, 7, 10, 11], and three disjunctions between Asia and the Neotropics [2, 7, 10]. The origin of these disjunctions has often been explained by hypotheses that invoke intercontinental connectivity via land bridges and corridors with suitable climates, with vicariance resulting from the disruption of wider distributions due to changing climatic conditions [4]. Such explanations often rely on the ‘boreotropics’ hypothesis [7], in which intercontinental biotic migrations were possible via tropical forests that extended into northern latitudes during the Late Palaeocene–Early Eocene thermal maximum (52–50 Mya), followed by disruption of this boreotropical vegetation as a result of climate deterioration in the late Eocene and a drastic temperature drop at the Eocene-Oligocene boundary [12, 13]. African-Asian disjunctions have also been explained either by overland dispersal during the Middle Miocene climatic optimum (17–15 Ma [13]) that coincided with land connections due to tectonic movements (the ‘Miocene geodispersal’ hypothesis: [9]); or by biotic ‘rafting’ on the Indian tectonic fragment prior to its collision with Asia during the Eocene [9, 14]. Although inherently immune to falsification, stochastic transoceanic dispersal events can also be invoked to explain intercontinental disjunctions [6, 15].

The present study focuses on the historical biogeography and associated evolutionary diversification of *Disepalum* and related genera within Annonaceae subfam. Annonoideae tribe Annoneae. A conspicuous Neotropical-Asian disjunction is apparent between the sister genera *Asimina* and *Disepalum*: *Asimina* is largely restricted to subtropical North America, although one species, *A. triloba*, extends into the temperate zone; whereas *Disepalum* is distributed in tropical and subtropical Southeast Asia [2, 7]. Previous ancestral area reconstructions have indicated a likely vicariance event between Africa and Asia at the Annoneae crown node [4]. These splits have been explained, largely based on broadly congruent molecular divergence time estimates, by invoking the Eocene ‘boreotropics’ hypothesis [7]. These underlying estimates of divergence times were based, however, on studies that included only a very limited number of *Asimina* and *Disepalum* accessions, a limitation that can result in severe underestimation of divergence times [16].

The first objective of this paper is therefore to use a more comprehensive sampling of taxa to re-evaluate whether the timing of the Neotropical-Asian disjunction in the *Asimina-Disepalum* clade is congruent with the disruption of boreotropical forests associated with post-Eocene climate change. Molecular divergence time estimation is undertaken here using extensive taxon sampling from the genera *Asimina* and *Disepalum*, with the implementation of uncorrelated relaxed molecular clock (UCLD) model and two fossil calibrations, viz. *Endressinia brasiliana* and *Futabanthus asamigawaensis*. Likelihood and Bayesian ancestral area estimations are employed to infer the direction of biotic exchange between the Old and New Worlds, as well as probable dispersal and vicariance events within the *Asimina-Disepalum* clade.

Molecular divergence times and ancestral area estimations in the *Asimina-Disepalum* clade may furthermore provide some insight into general biogeographic and diversification patterns in Southeast Asia. *Disepalum* consists of nine species of shrubs and trees inhabiting tropical lowland and montane forests at elevations from sea level to 2500 m in continental Southeast Asia and western Malesia [17]. *Disepalum* is divisible into two monophyletic subgenera [18]: (1) subgen. *Enicosanthellum* (three species), with a trimerous perianth consisting of a calyx of three sepals and two whorls of three petals; and (2) subgen. *Disepalum* (six species), with an aberrant floral structure comprising a calyx of two sepals and a single whorl of connate petals. The two subgenera occupy distinct geographical areas: subgen. *Enicosanthellum* is distributed in montane forests (600–1900 m altitude) in continental Asia, whereas subgen. *Disepalum* is largely confined to tropical lowland forests (generally sea level to 1000 m, although *D. platypetalum* Merr. extends to 2500 m) in western Malesia [17]. A previous study [17] explained the current ecological and geographical patterns observed in *Disepalum* by suggesting that continental Asian montane forests (encompassing the contemporary distribution of subgen. *Enicosanthellum*) are likely to represent the ancestral area and habitat for the genus, and that the genus might then have dispersed into western Malesia and diversified in lowland rainforests.

To investigate this hypothesis, we reconstruct ecological preferences for each extant species within the *Asimina-Disepalum* clade based upon their known current distribution in relation to topological and bioclimatic parameters using species distribution models. Since ecological preferences are heritable [19, and references therein], optimization of the value for each parameter at internal nodes enables niche parameters to be reconstructed across evolutionary time [19, 20]. Comparison of ecological tolerance among extant and ancestral species within the clade may enable insights in likely migration routes of lineages, as well as evaluating the role of bioclimatic ecological differentiation in the diversification of *Disepalum* within Southeast Asia.

## Methods

### Taxon sampling and DNA region selection

Single accessions representing 97 species were sampled. The ingroup consisted of eight *Disepalum* species (all species except *D. acuminatissium*, which is only known from fragmentary type material), and 41 species selected from six closely related genera within the tribe Annoneae, including *Annona*, *Anonidium*, *Asimina*, *Diclinanona*, *Goniothalamus* and *Neostenanthera*. The outgroup consisted of representatives from main clades across the family, of which 22 species were selected from 20 genera belonging to subfam. Annonoideae (other than the tribe Annoneae), 14 species from subfam. Malmeoideae, five from subfam. Ambavioideae, and two *Anaxagorea* species from subfam. Anaxagoreoideae. In order to calibrate the molecular clock for divergence time estimation, five representatives from four other families in the Magnoliales were selected, viz. Degeneriaceae (*Degeneria vitiensis*), Eupomatiaceae (*Eupomatia silvatica*), Himantandraceae (*Galbulimima belgraveana*), and Magnoliaceae (*Liriodendron chinense* and *Magnolia kobus*).

The final dataset included sequences of *Disepalum* species from four chloroplast DNA (cpDNA) regions (*mat*K, *trn*L-F, *ycf*1 and *ndh*F) and two nuclear DNA (nDNA) regions (*AP*3 and *phy*A) derived from our previous study [18], although the two nuclear regions were missing for *D. longipes* and one (*phyA*) was missing for *D. petelotii*. The dataset was supplemented with chloroplast data (*mat*K, *trn*L-F, *ycf*1 and *ndh*F) downloaded from the nucleotide database of the National Center for Biotechnology Information (http://www.ncbi.nlm.nih.gov). The relevant collection information, voucher specimens and GenBank accession numbers are listed in Additional file 1.

### Phylogenetic reconstruction and molecular divergence time estimation

Sequence data were assembled and edited using Geneious 5.4.3 [21]. They were then pre-aligned by the MAFFT [22] plugin in Geneious with automatic algorithm selection and default settings, followed by manual checking and optimization. A total of 108 ambiguously aligned positions within the non-coding region in the *trn*L-F region were excluded, resulting in a concatenated alignment consisting of 8278 characters. Our earlier phylogenetic study [18] included an assessment of potential incongruence between the cpDNA and nrDNA datasets using a visual inspection of topologies and by applying the incongruence length difference (ILD) test: no significant incongruence was detected.

Two fossils, *Endressinia brasiliana* and *Futabanthus asamigawaensis*, were used to calibrate the molecular phylogeny. *Endressinia brasiliana* is a fossilized flowering branch from the early Cretaceous (ca. 112 Mya) of Brazil [23], used to calibrate the Magnoliineae crown node (i.e., the root node of the phylogeny) [24, 25]. A lognormal prior distribution calibration (offset = 112; mean = 7.75; Log_(Stdev)_ = 13.4) was assigned, providing a distribution with a mean of 116.7 Mya (95% probability interval = 136.4–112 Mya). This soft upper bound of the distribution was defined with reference to the age of the earliest pollen grain fossils unequivocally assigned to the angiosperm crown group from the Hauterivian in the Early Cretaceous (136.4–130 Mya [26, 27]). *Futabanthus asamigawaensis* is a fossilized flower from the late Cretaceous (ca. 89 Mya) of Japan [28], which was used to calibrate the stem node of the clade including all Annonaceae subfamilies except the early divergent subfam. Anaxagoreoideae (i.e., the Annonaceae crown node). The phylogenetic position of *Futabanthus* is based on the absence of inner staminodes which is likely to be synapomorphic for *Futabanthus* and the Annonaceae excluding subfam. Anaxagoreoideae: the presence of inner staminodes in *Anaxagorea* (subfam. Anaxagoreoideae) and the outgroup taxa (including *Degeneria*, *Galbulimima* and *Eupomatia*) is likely to be plesiomorphic [28]. A lognormal prior distribution (offset = 89; mean = 3.4; Log_(Stdev)_ = 13.4) was applied, providing a distribution with a mean of 91.1 Mya (95% probability interval = 99.7–89 Mya), which assigns the highest probabilities to ages substantially older than the minimum age provided by the fossil dating.

Divergence times were estimated using BEAST 2.1.3 [29], with the dataset partitioned based on DNA region identity. Substitution models for each gene were incorporated according to the Akaike Information Criterion (AIC) in MrModelTest 2.3 [30]: the general time-reversible nucleotide substitution model with among-site variation modelled as a gamma distribution (GTR + Γ) was selected for the *mat*K, *trn*L-F, *ycf*1 and *AP*3 partitions; GTR + Γ and a proportion of invariable sites (I) was selected for the *ndh*F partition; and the Hasegawa-Kishino-Yano model with a proportion of invariable sites (HKY + I) was selected for the *phy*A partition. An uncorrelated lognormal-distributed (UCLD) relaxed molecular clock model was applied [31] and the Yule process was selected as tree prior [31, 32]. Eight independent Markov chain Monte Carlo (MCMC) analyses were performed in BEAST, each with 100 million generations sampled every 5,000th generation.

MCMC samples were analysed in Tracer 1.5 [33] to assess whether they were drawn from a stationary, unimodal distribution and whether adequate effective sample sizes (ESS) for each parameter had been reached (ESS > 200). The eight tree files were combined in LogCombiner 2.1.3 [29] with the burn-in set to 25%. Post-burn-in samples were summarised using maximum clade credibility (MCC) tree option in TreeAnnotator 2.1.3 [29] with mean age, 95% highest posterior density (HPD) intervals and posterior clade probabilities (PP) calculated for each node.

### Ancestral area reconstruction

Seven broadly defined biogeographical regions were delimited based upon the extant distribution of taxa in the tribe Annoneae: (A) North America; (B) South America; (C) continental Africa; (D) southern India and Sri Lanka; (E) continental Asia, including southern China, Indochina, Thailand, Myanmar and north-eastern India; (F) western Malesia, west of Wallace’s line, including Peninsular Malaysia, Sumatra, Java, Bali, Borneo and the Philippines; and (G) the Indo-Australian Archipelago east of Wallace’s line, including Sulawesi, the Moluccas, New Guinea, Australia, and some Pacific islands.

Ancestral area reconstruction was restricted to taxa in the tribe Annoneae, with all other taxa pruned from the input trees. A Bayesian approach to dispersal-vicariance analysis (S-DIVA) [34, 35] and a likelihood approach using the dispersal-extinction-cladogenesis (DEC) model [36, 37] were used, both implemented in RASP 3.2 [38, 39]. The post-burn-in trees from the BEAST analysis were resampled every 600,000 generations in LogCombiner 2.1.3 [29], yielding 1000 trees. Ancestral areas were reconstructed independently on these 1000 post-burn-in trees from the BEAST analysis. For both S-DIVA and DEC, the maximum number of ancestral areas at each node was constrained to two as ancestral ranges were assumed to be similar to the ranges of extant species. Values in the dispersal constraint matrix were set to 1. Relative probabilities of ancestral areas for each node were recorded and summarized on the MCC tree from the BEAST analysis.

### Species distribution modelling

#### Locality data

Locality data were compiled from at least three sources for each species of *Disepalum* and *Asimina* and an outgroup species, *Annona muricata*. Locality data were first obtained from the open-access online database of the Global Biodiversity Information Facility (GBIF; http://www.gbif.org/). These data points were supplemented by georeferenced herbarium records (taxonomic identities verified by the authors) from various herbaria (A, HKU, K, KEP, L, NY and S; herbarium abbreviations according to [40]). Herbarium specimens lacking precise coordinates were georeferenced using Google Earth (http://www.google.com/earth/) or the GeoNames geographical database (http://www.geonames.org/). Duplicates and ambiguous points were excluded manually.

‘Pseudo-occurrences’ were added for five *Asimina* species and two *Disepalum* species that had fewer than ten true occurrences (Additional file 1: Table S2) in order to reach the minimum number required for ecological niche modelling [41, 42]: for taxa with 5–9 known localities (*A. pygmaea* and *D. petelotii*), pseudo-occurrences were added 1 km to the east of randomly selected true locality points; for taxa with 2–4 known localities (*A. obovata*, *A. rugelii*, *A. speciosa* and *D. aciculare*), pseudo-occurrences were located 1 km to the east, west, north and south; for taxa with only one known locality (*A. pulchella*), pseudo-occurrences were added 1 km plus further 1 km to the east, west, north and south, and an extra pseudo-occurrence was added an additional 1 km to the east. The final dataset consisted of a total of 701 locality points for 18 species (one *Annona* species, nine *Asimina* species and eight *Disepalum* species), ranging from 10 to 256 locality points for each species. The number of true occurrences, pseudo-occurrences and total number of occurrences for each species for constructing the models are summarized in Additional file 1: Table S2. High values for the Area under the Receiver Operating Characteristic curve (AUC), ranging from 0.95 to 1.00, were obtained for all the species including those with pseudo-occurrences as indicated in Table S4, showing good predictive performance, similar to the observed geographical ranges.

#### Environmental data

Twenty topological and bioclimatic variables, including altitude and 19 climate measurements (enumerated in the caption to Table 1) that summarise current environmental temperatures and precipitation, were downloaded from WorldClim (http://www.worldclim.org [43]) at 30-arc-seconds spatial resolution (equivalent to ca. 1 × 1 km at the equator). The maps covering the relevant areas were downloaded and trimmed before analysis. The value for each parameter for every unique locality point was extracted using the R package dismo [44].

**Table 1:** Topological and bioclimatic variables considered for niche modelling in this study, with mean AUC values and correlation coefficients for each variable pair

Variable	Mean AUC	ALT	Bio1	Bio2	Bio3	Bio4	Bio5	Bio6	Bio7	Bio8	Bio9	Bio10	Bio11	Bio12	Bio13	Bio14	Bio15	Bio16	Bio17	Bio18
ALT^a^	0.79																			
Bio1	0.77	−0.08																		
Bio2^a^	0.81	−0.16	−0.54																	
Bio3	0.85	0.31	**0.83**	−0.63																
Bio4	0.84	−0.31	**−0.89**	0.66	**−0.96**															
Bio5^a^	0.87	−0.79	0.17	0.46	−0.29	0.27														
Bio6	0.83	0.11	**0.96**	−0.68	**0.93**	**−0.97**	−0.09													
Bio7^a^	0.91	−0.28	**−0.88**	0.75	**−0.95**	**0.99**	0.31	**−0.97**												
Bio8^a^	0.82	−0.21	0.60	−0.40	0.41	−0.47	0.19	0.55	−0.48											
Bio9	0.81	0.01	**0.89**	−0.45	0.77	**−0.83**	0.07	**0.87**	**−0.82**	0.27										
Bio10	0.84	−0.77	0.55	0.02	0.07	−0.11	**0.87**	0.32	−0.11	0.43	0.42									
Bio11	0.82	0.11	**0.98**	−0.62	**0.92**	**−0.97**	−0.04	**1.00**	**−0.96**	0.55	**0.89**	0.35								
Bio12^a^	0.86	0.18	0.59	−0.61	0.73	−0.68	−0.25	0.68	−0.70	0.22	0.59	0.06	0.65							
Bio13	0.83	0.27	0.65	−0.59	0.71	−0.73	−0.22	0.72	−0.74	0.32	0.60	0.08	0.71	**0.88**						
Bio14	0.89	−0.06	0.25	−0.39	0.43	−0.29	−0.18	0.32	−0.35	−0.01	0.33	0.04	0.28	0.71	0.36					
Bio15^a^	0.87	0.24	0.46	−0.16	0.32	−0.46	0.03	0.43	−0.40	0.42	0.31	0.14	0.46	−0.02	0.38	−0.57				
Bio16	0.83	0.26	0.66	−0.60	0.72	−0.74	−0.23	0.73	−0.75	0.33	0.61	0.08	0.72	**0.91**	**0.99**	0.41	0.34			
Bio17^a^	0.93	−0.06	0.28	−0.41	0.45	−0.31	−0.17	0.35	−0.37	−0.01	0.35	0.06	0.31	0.74	0.38	**0.99**	−0.56	0.43		
Bio18^a^	0.84	0.25	0.52	−0.56	0.59	−0.60	−0.32	0.59	−0.63	0.35	0.45	0.02	0.57	0.72	0.68	0.45	0.18	0.72	0.47	
Bio19	0.87	−0.01	0.42	−0.42	0.56	−0.45	−0.10	0.48	−0.49	0.08	0.47	0.12	0.45	**0.84**	0.67	0.77	−0.25	0.69	0.79	0.40

*Abbreviations*: *ALT* Altitude, *Bio1* Annual mean temperature, *Bio2* Mean diurnal range (Mean of monthly (max temperature – min temperature)), *Bio3* Isothermality (Bio2/Bio7 × 100), *Bio4* Temperature seasonality (standard deviation × 100), *Bio5* Maximum temperature of warmest month, *Bio6* Minimum temperature of coldest month, *Bio7* Temperature Annual Range (Bio5–Bio6), *Bio8* Mean temperature of wettest quarter, *Bio9* Mean temperature of driest quarter, *Bio10* Mean temperature of warmest quarter, *Bio11* Mean temperature of coldest quarter, *Bio12* Annual precipitation, *Bio13* Precipitation of wettest month, *Bio14* Precipitation of driest month, *Bio15* Precipitation seasonality (coefficient of variation), *Bio16* Precipitation of wettest quarter, *Bio17* Precipitation of driest quarter, *Bio18* Precipitation of warmest quarter, *Bio19* Precipitation of coldest quarter. Temperature is given in °C, precipitation in mm. Variables used to construct ecological niche models are indicated with^a^. Correlation coefficients ≥0.8 are indicated using bold font

In order to avoid over-parameterization of analyses, a Pearson correlation test was performed in SPSS Statistics 17.0 [45], identifying pairs of variables that were highly correlated (coefficient ≥ 0.8 [46]). For pairs of highly correlated variables, the method described by Töpel et al. [47] was applied to select the variable with greater predictive power. AUC values were acquired by constructing niche models for all 18 species with each of the 20 variables independently [47] using the maximum entropy method in Maxent 3.3.3 [48]. The AUC values measure the degree to which the model differs from random: values range from 0 to 1, in which a value of 0.5 indicates a random niche prediction, whereas values approaching 1 denote well-performing models [49]. For highly correlated variables (correlation coefficient ≥ 0.8) the mean AUC value was calculated for each variable and the variable with the lower value was excluded.

Nine environmental variables were chosen for subsequent analyses, including: altitude (ALT); mean diurnal temperature range (Bio2); maximum temperature of the warmest month (Bio5); annual temperature range (Bio7); mean temperature of the wettest quarter (Bio8); annual precipitation (Bio12); coefficient of variation of precipitation seasonality (Bio15); precipitation of the driest quarter (Bio17); and precipitation of the warmest quarter (Bio18). The mean AUC values for all topological and bioclimatic variables and correlation coefficients for variable pairs are presented in Table 1.

#### Present-day species distribution model building and testing

We used Maxent 3.3.3 [48] for species distribution model building and testing. Maxent uses an algorithm for maximum entropy to estimate the potential niche of species and the probability of occurrence [48]. This approach has been shown to work well compared to other methods [50], particularly when relatively few locality points are available [35, 51, 52].

To construct the models, 75% and 25% of the locality points of each species are randomly assigned to training and testing datasets, respectively. All models were run using auto-features in logistic format, with a maximum of 500 iterations and regularization multiplier of 1.0. The importance of individual environmental variables in explaining the distribution of each species modelled was determined by running jackknife tests within the Maxent interface. AUC estimation is used to measure the degree to which models differ from random, with those with AUC > 0.7 regarded as reliable [3, 53].

#### Ancestral niche parameter value reconstruction and ancestral niche model building

The mean, standard deviation, minimum and maximum values for the nine selected bioclimatic parameters were calculated based on the current distribution data for extant species. Each value was independently optimized in Mesquite 2.7.4 [54] across the MCC tree from the BEAST analysis, from which all taxa were pruned except those sampled for niche modelling. The values were treated as continuous characters and square-change parsimony optimization was implemented.

BIOCLIM [55] was used to build bioclimatic niche models at each node within the *Asimina*-*Disepalum* clade with a posterior probability (PP) value exceeding 0.95 using the maximum and minimum parameter values reconstructed at internal nodes. BIOCLIM defines the climatic niche of a given species using the observed values for each bioclimatic variable, and predicts the potential species distribution by calculating the probability of species occurrence for each cell of the map [56, 57]. The models were projected onto present-day climatic data, enabling a visual comparison of ecological preferences of extant taxa and reconstructed ancestral niches.

#### Niche comparisons

In order to assess the degree of ecological differentiation between sister lineages, the Maxent outputs for each pair of species were compared using the Schoener’s [58] *D* and *I* statistics in ENMTools [59] to quantify and compare niche similarity. The similarity indices were calculated by comparing differences in habitat suitability for each grid cell between bioclimatic niche models [59]. Both indices are summed from 0 to 1, indicating the spectrum between no overlap between niches at all and completely identical niches. Niche comparisons were also made between taxa using axes acquired from detrended component analyses (DCA) of the nine selected environmental variables using the statistical package PAST [60].

## Results

### Phylogenetic reconstruction and molecular divergence time estimation

**Fig. 1:**
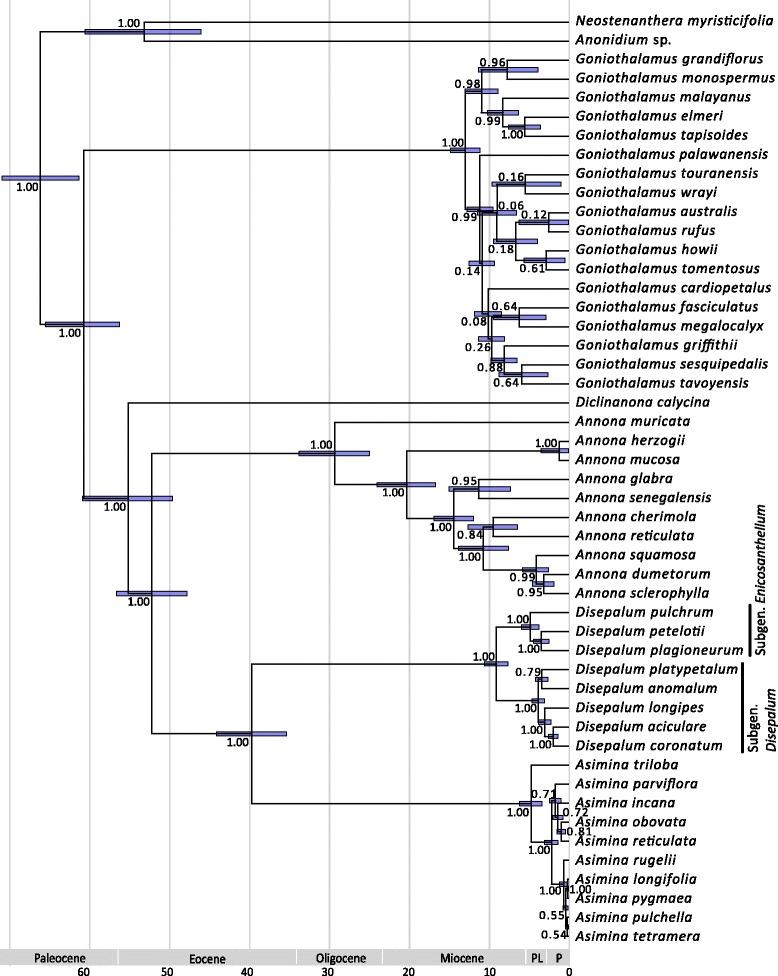
Chronogram of the tribe Annoneae extracted from the maximum clade credibility (MCC) tree from the BEAST analysis of the Annonaceae. Nodes are posterior mean ages (Mya) with blue node bars representing the 95% HPD intervals. Numbers at nodes indicate Bayesian posterior probabilities (PP)

The MCC tree from the BEAST analysis reveals four strongly supported clades (PP = 1.00) within the Annonaceae, corresponding to the four subfamilies, Anaxagoreoideae, Ambavioideae, Malmeoideae and Annonoideae. The topology of the tribe Annoneae (PP = 1.00; Fig. 1) is consistent with the results of our previous molecular phylogenetic analyses [18], confirming the sister relationship between the *Anonidium-Neostenanthera* and the other genera in the tribe. The sister relationship between *Asimina* and *Disepalum* is strongly supported (PP = 1.00; Fig. 1). Clades representing *Disepalum* subgenera *Disepalum* and *Enicosanthellum* are consistently retrieved, with all nodes well resolved and strongly supported (PP = 1.00; Fig. 1) except for the relationship between *D. platypetalum* and other representatives of subgen. *Disepalum*, in which the sister relationship between *D. anomalum* and *D. platypetalum* is only weakly supported (PP = 0.76; Fig. 1).

The mean rate of evolution is inferred as 7.1 × 10^−4^ substitutions per site per million years (95% HPD = 6.3 × 10^−4^–8.0 × 10^−4^), with the birth rate (net speciation rate indicated by Yule prior) at 0.04 per million years (95% HPD = 0.031–0.048). The mean divergence time estimates, 95% HPD intervals and PP values of nodes of interest are summarized in Table 2 and mapped onto the MCC chronogram of the tribe Annoneae (Fig. 1). The three intercontinental vicariance events (described below) occurred between the early Palaeocene and Eocene, with mean ages of 66.5 Mya (95% HPD = 71.3–61.6 Mya; node 97), 61.0 Mya (95% HPD = 65.6–56.5 Mya; node 96), and 40.0 Mya (95% HPD = 44.3–35.5 Mya; node 93).

**Table 2:** Posterior age distributions of major nodes in the Annonaceae and *Disepalum* based on BEAST analyses, with results of ancestral range reconstructions using statistical dispersal-vicariance analysis (S-DIVA) and likelihood estimation under the dispersal-extinction-cladogenesis (DEC) model2

	Bayesian PP	Age estimates (Mya)		Ancestral area			
Node		Mean	(95% HPD)	S-DIVA		DEC	
C1: Magnoliaceae stem	1.00	115.7	(119.9–112.6)				
C2: Annonaceae crown	1.00	99.3	(104.9–94.0)				
Subfam. Ambavioideae stem	1.00	93.6	(99.2–88.3)				
Subfam. Ambavioideae crown	1.00	40.0	(46.6–33.2)				
Subfam. Malmeoideae stem	1.00	92.0	(97.5–86.7)				
Subfam. Malmeoideae crown	1.00	34.6	(41.9–27.5)				
Subfam. Annonoideae crown	1.00	89.3	(94.8–84.0)				
Tribe Annoneae stem	1.00	71.0	(76.1–66.2)				
97: Tribe Annoneae crown	1.00	66.5	(71.3–61.6)	BC	(48.3)	CF	(53.4)
				CF	(48.1)	BC	(30.1)
				CG	(3.6)	BF	(8.5)
						CG	(7.3)
50: *Anonidium-Neostenanthera* crown	1.00	53.4	(60.8–46.2)	C	(100.0)	C	(85.8)
						CF	(14.2)
96: *Goniothalamus* stem	1.00	61.0	(65.6–56.5)	BF	(48.1)	CF	(46.9)
				F	(48.1)	BC	(25.4)
				BG	(3.7)	BF	(18.5)
						CG	(9.2)
95: *Diclinanona* crown	1.00	55.4	(61.6–50.2)	B	(50.1)	BF	(45.0)
				BF	(49.9)	B	(36.9)
						F	(18.2)
94: *Asimina-Disepalum* stem	1.00	52.5	(56.9–48.1)	BF	(50.0)	BF	(47.9)
				AB	(50.0)	B	(34.0)
						F	(18.2)
93: *Asimina-Disepalum* crown	1.00	40.0	(44.3–35.5)	AF	(100.0)	F	(34.1)
						AF	(25.9)
						BF	(15.1)
						B	(7.6)
						AB	(6.7)
						BE	(5.8)
						EF	(4.9)
83: *Disepalum* crown	1.00	9.1	(10.6–7.6)	F	(100.0)	EF	(62.5)
						F	(37.5)
82: Subgen. *Disepalum* crown	1.00	3.8	(4.6–3.0)	F	(100.0)	F	(100.0)
81	1.00	3.0	(3.8–2.2)	F	(100.0)	F	(100.0)
80	1.00	1.9	(2.5–1.3)	F	(100.0)	F	(100.0)
79	0.79	3.3	(4.1–2.6)	F	(100.0)	F	(100.0)
78: Subgen. *Enicosanthellum* crown	1.00	4.8	(5.9–3.7)	EF	(100.0)	EF	(100.0)
77	1.00	3.4	(4.4–2.4)	E	(100.0)	E	(100.0)

**Fig. 2:**
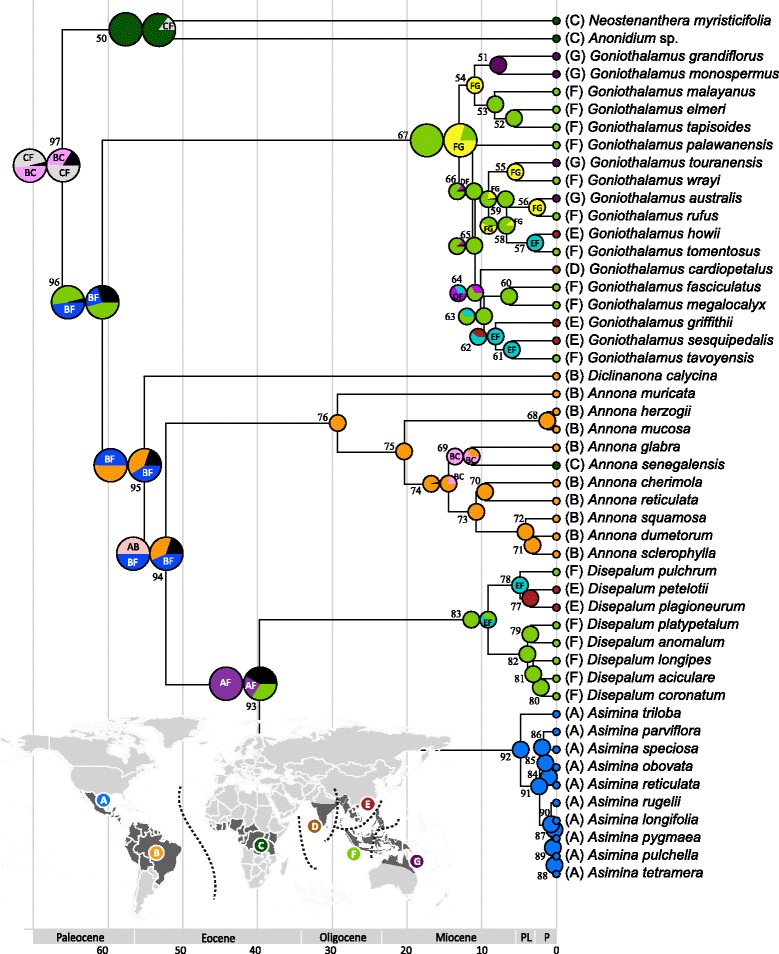
Ancestral area optimization by S-DIVA and DEC model in the tribe Annoneae. Pie charts at nodes represent relative frequencies of ancestral area reconstructions using S-DIVA (left) and DEC (right) optimizations; single pie chart is shown for congruent S-DIVA and DEC results. Colours in the pie charts correspond with geographical areas (see inset map) except where superimposed with letters indicating broader ancestral areas; only two areas with the highest frequency are shown, while *black* is used to represent aggregated minor explanations. Numbers beside pie charts represent node numbers. Geological epoch abbreviations: PL = Pliocene; P = Pleistocene. Inset: Biogeographical areas used in the S-DIVA optimization. A: North America; B: South America; C: Africa excluding Madagascar; D: Southern India and Sri Lanka; E: continental Asia including southern China, Indochina, Thailand, Myanmar and north-eastern India; F: western Malesia; and G: Southeast Asia and Australasia, east of Wallace’s line

Node numbers correlate with those shown in Fig. 2. Fossil calibrations: C1 = *Endressinia brasiliana*; C2 = *Futabanthus asamigawaensis*

### Ancestral area reconstruction

The ancestral areas inferred by S-DIVA and likelihood estimation under the DEC model are presented as Fig. 2, with details of inferred dispersal and vicariance events at nodes of interest provided in Table 2. Three intercontinental vicariance events are identified in the tribe: (1) between the Neotropics and Africa (divergence between the *Anonidium*-*Neostenanthera* and *Annona*-*Asimina*-*Diclinanona*-*Disepalum-Goniothalamus* lineages; node 97); (2) between the Neotropics and western Malesia and/or regions east of Wallace’s line (divergence between the *Annona*-*Asimina*-*Diclinanona*-*Disepalum* clade and *Goniothalamus*; node 96); and (3) between the Neotropics and Asia (divergence between *Asimina* and *Disepalum*; node 93). The ancestral range of the *Annona-Asimina*-*Disepalum* clade (node 94) was inferred as Neotropical or Neotropical-Asian (S-DIVA: BF = 50%, AB = 50%; DEC: BF = 47.9%, B = 34.0%, F = 18.2%) with dispersal from the Neotropics to Asia indicated between the stem and crown nodes of the clade (nodes 94 and 93).

The ancestral range at the stem node of *Disepalum* (node 93 in Table 2 and Fig. 2) is estimated as the Neotropics and western Malesia, or western Malesia (S-DIVA: AF = 100%; DEC: F = 34.1%, AF = 25.9%, BF = 15.1%, B = 7.6%, AB = 6.7%, BE = 5.8%, EF = 4.9%). Reconstructions within *Disepalum* indicate dispersal from western Malesia to continental Asia, followed by a vicariance event within subgen. *Enicosanthellum* (i.e., a split between *D. pulchrum* and the *D. petelotii-plagioneurum* clade: S-DIVA and DEC: EF = 100%; node 78). The ancestral area of subgen. *Disepalum* is consistently retrieved as western Malesia (S-DIVA and DEC: F = 100%; node 82).

### Ecological niche and phyloclimatic modelling

**Fig. 3:**
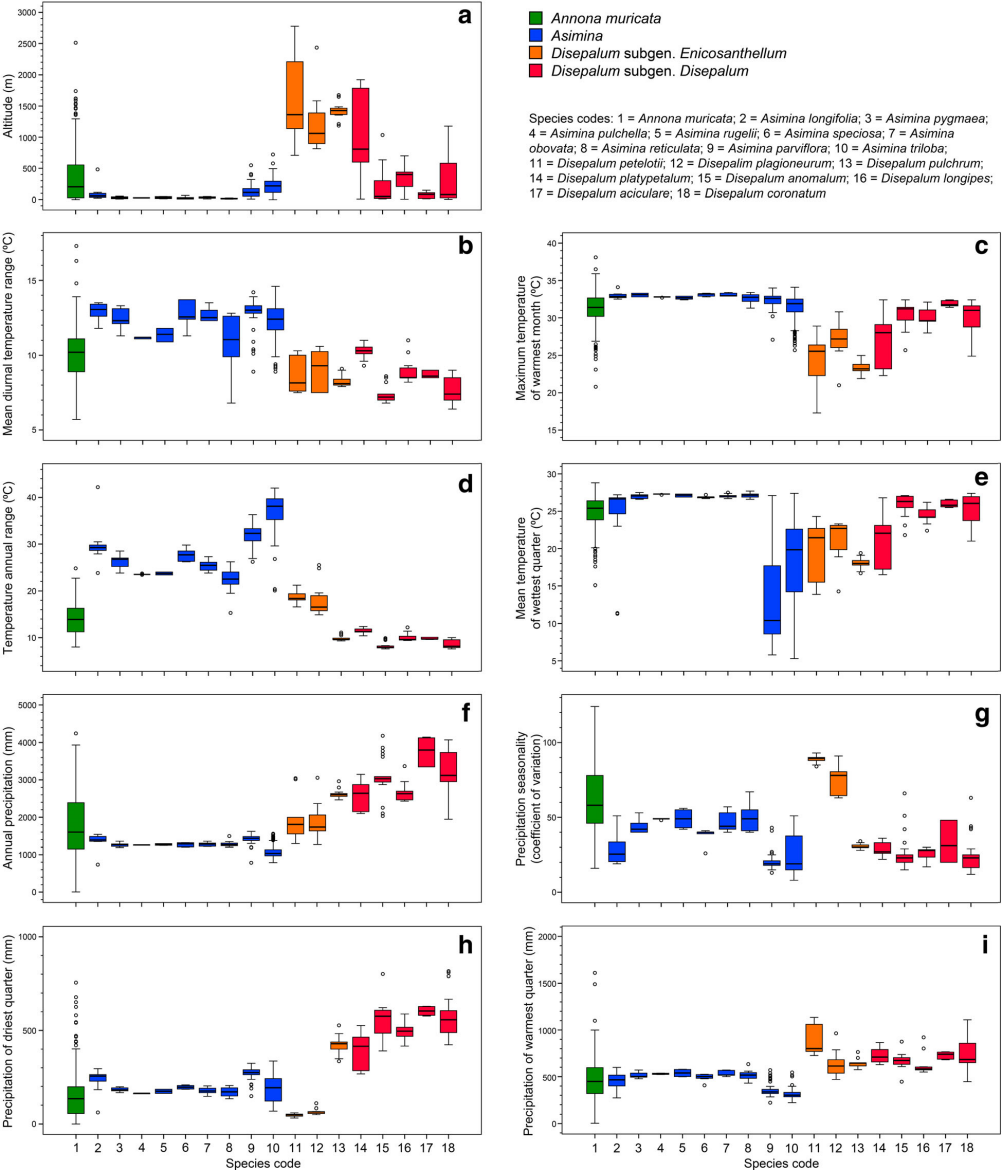
Climatic variation in selected species. **a** Altitude. **b** Bio2: mean diurnal temperature range (mean of monthly temperature [maximum temperature – minimum temperature]). **c** Bio5: maximum temperature of warmest month. **d** Bio7: temperature annual range (Bio5 – Bio6). **e** Bio8: mean temperature of wettest quarter. **f** Bio12: annual precipitation. **g** Bio 15: precipitation seasonality (coefficient of variation). **h** Bio 17: precipitation of driest quarter. **i** Bio18: precipitation of warmest quarter. Sample sizes: *Annona muricata* (*n* = 200); *Asimina longifolia* (*n* = 12); *A. pygmaea* (*n* = 10); *A. pulchella* (*n* = 10); *A. rugelii* (*n* = 10); *A. speciosa* (*n* = 10); *A. obovata* (*n* = 10); *A. reticulata* (*n* = 10); *A. parviflora* (*n* = 49); *A. triloba* (*n* = 256); *Disepalum petelotii* (*n* = 10); *D. plagioneurum* (*n* = 12); *D. pulchrum* (*n* = 16); *D. platypetalum* (*n* = 16); *D. anomalum* (*n* = 21); *D. longipes* (*n* = 11); *D. aciculare* (*n* = 10); *D. coronatum* (*n* = 28)

The calculated mean values of the nine bioclimatic variables selected for the 18 species sampled in the ecological niche modelling are summarized (Additional file 1: Table S3), with boxplots showing the variation in species (Fig. 3). AUC values for the Maxent models range from 0.95 to 1.00, indicating good predictive performance (Additional file 1: Table S4). Altitude (ALT) is an important variable, significantly contributing to niche models of all *Asimina* species except *A. parviflora* and *A. triloba*, for which precipitation of the driest quarter (Bio17) and annual temperature range (Bio7), respectively, are the major contributing variables.

Altitude has only a minor impact on the models of *Disepalum* species (with the exception of *D. pulchrum* in which it contributes 23.2%), whereas annual temperature range (Bio7)—which is strongly correlated with the excluded temperature seasonality variable (Bio4)—substantially contributes to the models of all *Disepalum* species. Specific climatic variables form significant contributions to the ecological models of individual species, viz.: annual precipitation (Bio12) for *D. aciculare*; precipitation of the driest quarter (Bio17) for *D. coronatum*; and precipitation of the wettest quarter (Bio18) for the sister species *D. petelotii* and *D. plagioneurum*.

**Fig. 4:**
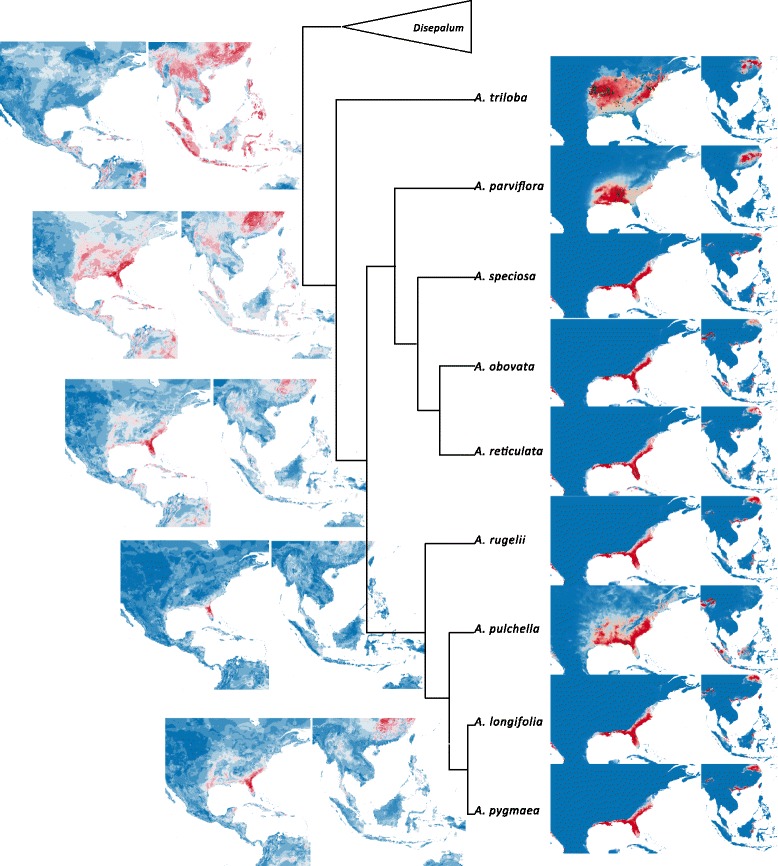
Niche models for Neotropical *Asimina* species derived using Maxent and ancestral reconstruction of bioclimatic envelopes by BIOCLIM. Projections of potential distribution of each species in south-eastern North America as well as Southeast Asia are presented for visual comparison with *Disepalum* (Fig. 5). Projections of bioclimatic tolerance of ancestral nodes on present-day climate of Southeast Asia are presented accordingly in the phylogeny. Only bioclimatic models for nodes with Bayesian posterior probability ≥0.95 are projected. See [76] for distributions of extant *Asimina* species

**Fig. 5:**
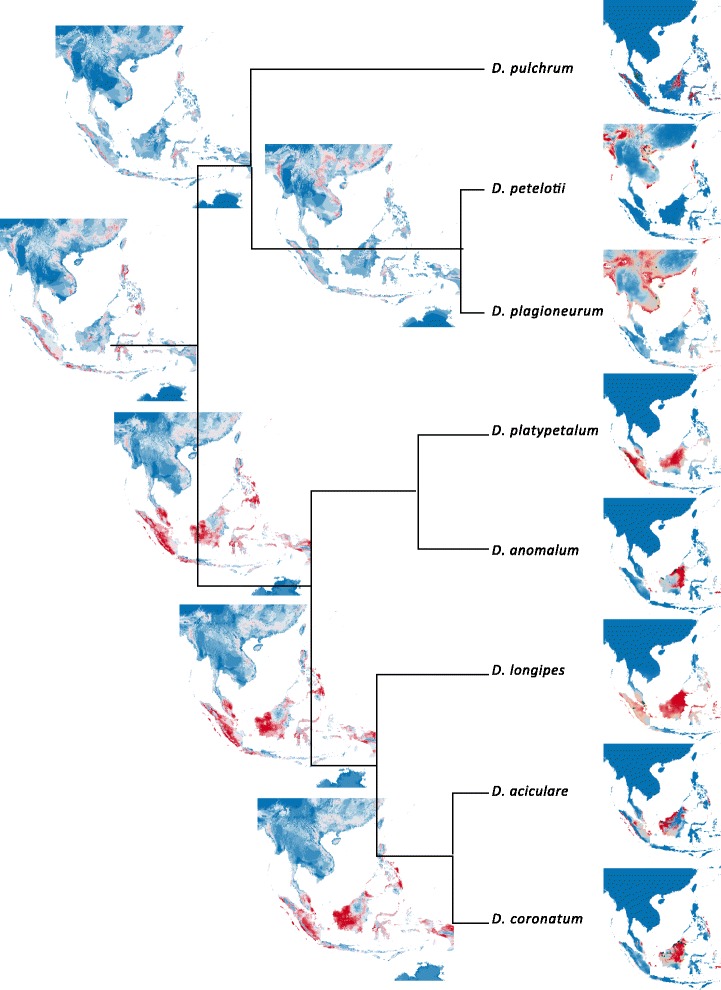
Niche models for *Disepalum* species derived using Maxent and ancestral reconstruction of bioclimatic envelopes by BIOCLIM. Projections of bioclimatic tolerance of ancestral nodes on present-day climate of Southeast Asia are presented in the phylogeny. Only bioclimatic models for nodes with Bayesian posterior probability ≥0.95 are projected. See [17] for distributions of extant *Disepalum* species

Maxent projections of potential distributions of extant *Asimina* species correspond closely to current species distributions in subtropical eastern North America (Fig. 4), although several regions in subtropical China are also indicted to possess ecological niches that would potentially be suitable for *Asimina* species. Two major regions in Asia are shown to have ecologically suitable habitats for *Disepalum* species (Fig. 5): *D. petelotii* and *D. plagioneurum* are projected onto subtropical continental Asia, emphasizing generally more seasonal climates; whereas the other *Disepalum* species (including *D. pulchrum* and species in subgen. *Enicosanthellum*) are projected onto perennially wet tropical regions in western Malesia.

Projections of reconstructed ancestral bioclimatic models using BIOCLIM onto present-day climates in Asia are shown in Figs. 4 and 5, with the optimized mean values for nodes provided in Additional file 1: Table S5. Ancestral niche modelling with reference to present-day climates indicates that the ancestor of the *Asimina-Disepalum* clade may have had a wide climatic tolerance, encompassing conditions found in current subtropical and tropical upland and montane habitats in Southeast Asia (Fig. 4). The bioclimatic model of the crown node of the *Asimina* clade is projected onto essentially subtropical regions of continental Asia (Fig. 4), whereas the model for the *Disepalum* crown node is projected mainly onto upland and montane areas in tropical Southeast Asia (Fig. 5). The projection of the niche model for the *Disepalum* subgen. *Disepalum* crown node indicates adaptation to climatic conditions similar to current conditions found in perennially wet tropical Malesia. In contrast, the projection of the niche model for the *Disepalum* subgen. *Enicosanthellum* crown node indicates a wider climatic range, encompassing both subtropical and tropical regions. Within subgen. *Enicosanthellum*, there is a clear split: the projection of the reconstructed bioclimatic model at the *D. petelotii-D. plagioneurum* crown node is similar to the current distribution of the two species in continental Southeast Asia, whereas *D. pulchrum* is restricted to perennially wet regions in Malesia.

The *D* and *I* statistics indicate strong niche similarity between species (Additional file 1: Table S6 and Additional file 1: Table S7). In general, *Asimina* has more consistent bioclimatic niche preferences than *Disepalum*, indicated by higher average *D* and *I* values (*Asimina*: *D*_mean_ = 0.44, *I*_mean_ = 0.63; *Disepalum*: *D*_mean_ = 0.28, *I*_mean_ = 0.49).

**Fig. 6:**
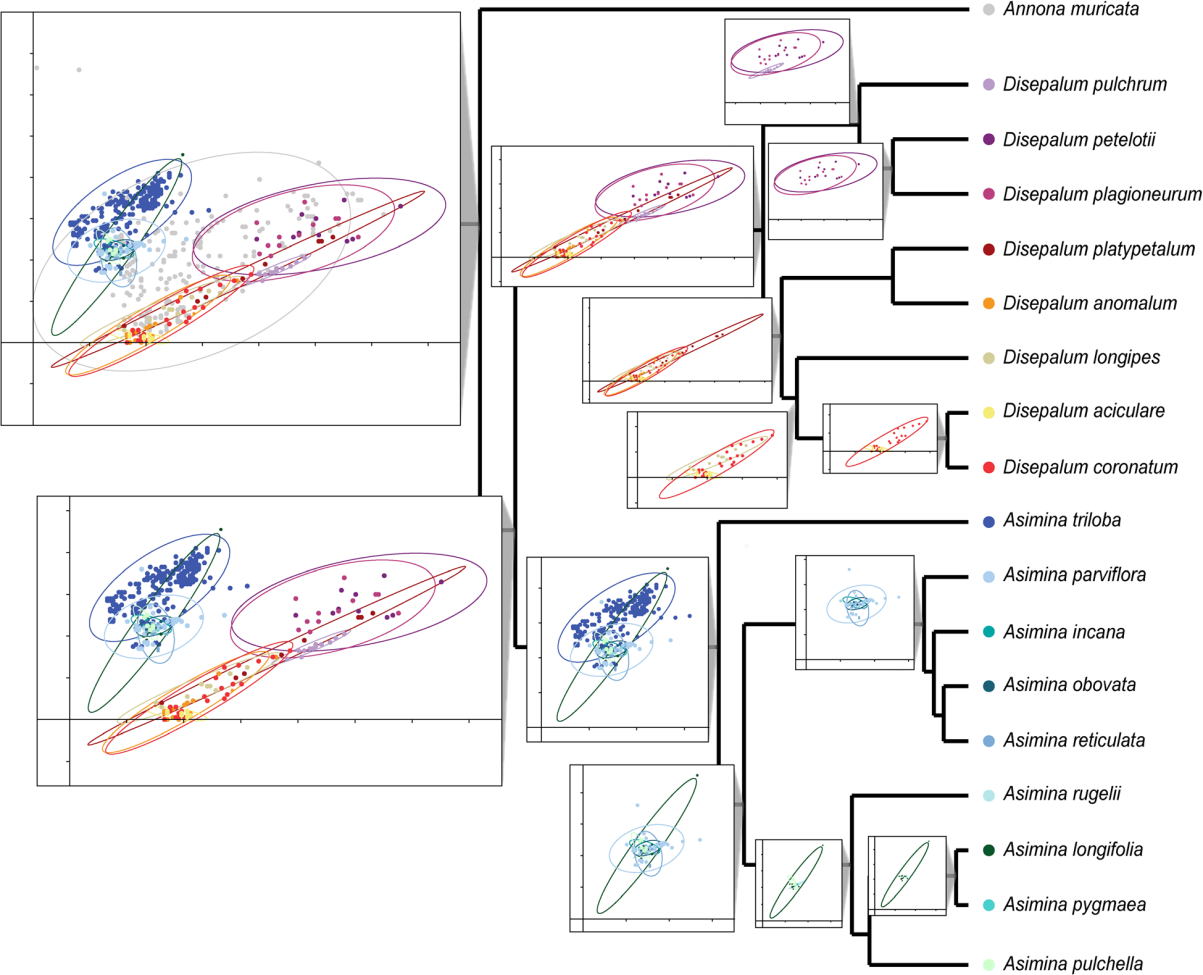
Plots obtained from detrended component analyses (DCA) based on the selected environmental variables in this study. Plots at each node display principal component results for all species within that lineage, and do not show reconstructed ancestral values. *Grey*: *Annona muricata*; *blue*: *Asimina* species; *purple*: species in subgen. *Enicosanthellum*; *orange to yellow*: species in subgen. *Disepalum*

The detrended components analysis (DCA) indicates overlap of bioclimatic preferences between sister lineages. DCA plots showing bioclimatic niche comparisons within lineages are displayed on the MCC tree from the BEAST analysis (Fig. 6), with the DCA plots at internal nodes representing all species included in that clade rather than ancestral reconstructions for the node. The first component describes a gradient corresponding to variation in altitude (ALT), maximum temperature of the warmest month (Bio5), and mean temperature of wettest quarter (Bio8); higher values of the altitude loads positively, whereas higher temperatures load negatively. The second component depicts a gradient of diurnal temperature (Bio2), annual temperature range (Bio7), annual precipitation (Bio12), coefficient of variation of precipitation seasonality (Bio15), precipitation of the driest quarter (Bio17), and precipitation of the warmest quarter (Bio18). More variable temperatures and greater seasonal precipitation values load positively, whereas higher precipitation values load negatively.

## Discussion

### Neotropical-Asian intercontinental disjunction in the tribe Annoneae

Estimation of mean divergence times indicates that the stem of the *Asimina-Disepalum* clade originated ca. 52.5 Mya (95% HPD = 56.9–48.1 Mya; Fig. 1) during the early to middle Eocene. S-DIVA and DEC analyses (Fig. 2) suggest that the ancestral area of the lineage was in the New World, with a dispersal event inferred from the New World to Asia between the stem and crown nodes. The timing of this dispersal event is congruent with the end of the Late Palaeocene-Early Eocene thermal maximum, which peaked in the Early Eocene optimum, ca. 52–50 Mya [13]. During this warming period, during which temperatures increased by ca. 6 °C over 20 million years [13], tropical forests expanded northwards and occupied regions from the equator to mid-latitudes across all northern continents [12, 61, 62]. Although the geographical extent of the expansion was variable it has been suggested that boreotropical forests are likely to have extended beyond 50° N in Europe [12]. The existence of the extensive boreotropical forests and the North America land bridges connecting the Old and the New Worlds (including the ‘Beringian’ connection between North America and East Asia, ca. 58 Mya; and the ‘Thulean’ route connecting North America and Europe via Greenland, ca. 57 Mya and 55.8 Mya: [63]) enabled intercontinental biotic exchange of tropical forest taxa in both directions (e.g., Lauraceae [64], Malpighiaceae [65], Meliaceae [66] and Rubiaceae [67]).

The fall in global temperatures around the Eocene-Oligocene boundary constricted and fragmented boreotropical forests, resulting either in the extinction of northern mid-latitude tropical vegetation or their migration southwards to tropical regions [2, 7, 61, 68]. The divergence time between the sister genera *Asimina* and *Disepalum* is estimated at ca. 40.0 Mya (95% HPD = 44.3–35.5 Mya; Fig. 1) during the middle to late Eocene, with vicariance resulting in two genera restricted to the Neotropics (*Asimina*) and Asia (*Disepalum*), respectively (Fig. 2). The divergence time estimate is congruent with the boreotropics hypothesis, i.e. that climatic deterioration during the Late Eocene and a sharp temperature drop at the Eocene-Oligocene boundary likely caused this intercontinental tropical disjunction. A broad climatic tolerance is inferred for the ancestral node of the *Asimina-Disepalum* clade, close to the current climate in tropical to subtropical Asia (Fig. 3). The ancestor of the *Asimina* lineage was probably adapted to a more seasonal climate in mid-latitudes, although the Annonaceae generally show a high degree of climatic niche conservatism in tropical and subtropical forests [3, 4]. The *Asimina* lineage initially diversified within subtropical regions of North America during the Pliocene (Fig. 2). Fossil seeds of *Asimina brownii* P.W. Thomson (resembling those of *A. parviflora* and *A. triloba*) have been recovered from early Miocene to Pliocene strata of Germany and France derived from tropical to subtropical swamp vegetation [69–71], suggesting that the lineage may have perpetuated in Europe, becoming extinct later. Our niche modelling (Fig. 4) demonstrates that extant *Asimina* species have similar climatic preferences to their ancestor, although this is expected as they share similar geographical distributions.

### Origin and diversification of the genus *Disepalum*

The most recent common ancestor (MRCA) of *Disepalum* is inferred to have had a wider distribution, either in western Malesia and continental Southeast Asia (DEC; Fig. 2) or exclusively in western Malesia (S-DIVA; Fig. 2). Optimal reconstructions of bioclimatic niches indicate that the MRCA of the *Disepalum* lineage was probably tolerant of tropical and subtropical climates (Fig. 5). It is likely that the decrease of global temperature around the Eocene-Oligocene boundary resulted in a southwards shift in ancestral *Disepalum* species towards subtropical and tropical regions of Southeast Asia, eventually becoming restricted to tropical Malesia.

The genus subsequently diverged into two clades, representing the subgenera *Disepalum* and *Enicosanthellum*. Western Malesia and continental Southeast Asia are collectively inferred as the most likely ancestral areas for the latter subgenus, with a subsequent vicariance event between western Malesia (*D. pulchrum*) and continental Asia (the *D. petelotii-D. plagioneurum* lineage) during the late Miocene to Pliocene (ca. 4.8 Mya; Fig. 2). This is congruent with the optimized niche models within the clade (Fig. 5), revealing a transition in bioclimatic tolerance from wetter environments to climates with more seasonal precipitation in the *D. petelotii-D. plagioneurum* lineage (Fig. 3). The ancestral area of subgen. *Disepalum* is inferred to be western Malesia (Fig. 2), with subsequent diversification within the region during the Pliocene.

### Role of ecological differentiation during diversification of *Disepalum*

Considerable ecological conservatism has previously been identified in the Annonaceae [2, 3], with most species restricted to tropical lowland forests. In contrast, *Disepalum* demonstrates significant altitudinal variation between species, ranging from sea level to 2500 m. Ecological and geographical patterns have previously been articulated by Johnson [17], who suggested that *Disepalum* initially diversified in montane forests in continental Asia and subsequently dispersed into and radiated within the lowland forests of western Malesia. Although our Maxent analyses (Fig. 5) suggest that altitude contributes relatively little to species distributions within *Disepalum*, the DCA plots (Fig. 6) indicate otherwise.

The seasonality of temperature and precipitation, which form major contributions to the first two detrended principal components, appears to greatly influence the geographical range of species. Seasonality directly corresponds with latitude, with more seasonal climates in more northerly latitudes. A strong divergence of climate niche preferences is identified in *Disepalum*, associated with the geographical separation of *D. pulchrum* (lower latitudes in Peninsular Malaysia) from other members of the subgen. *Enicosanthellum* lineage (higher latitudes in continental Asia). The broad ancestral climatic niche preferences of species in subgen. *Enicosanthellum*—ranging from tropical to subtropical present-day climates—together with the geographical separation between lineages suggest that climate niche differentiation was more likely to be a result of geographical isolation rather than a major driver of speciation in *Disepalum*. This finding is consistent with the inference of limited ecological divergence between species in the African Annonaceae genera *Isolona* and *Monodora* [3]. Other biotic or abiotic factors clearly need to be considered as potential drivers of diversification.

The bioclimatic tolerance of species in subgen. *Disepalum* appears to have been conserved, as it is similar to the current climates in tropical Asia, with consistently high annual rainfall and constant temperatures throughout the year. Factors that may have contributed to diversification within the subgenus include climate-driven sea level changes during the Pliocene and Pleistocene. The current islands of the Sunda Shelf, including Sumatra and Borneo, were connected with each other and with continental Southeast Asia during part of the Pliocene and most of the Pleistocene, interrupted by relatively short phases of high eustatic sea-levels [72–75]. This would have enabled overland dispersal of species between regions in western Malesia that are now isolated, including Peninsular Malaysia, Sumatra and Borneo. This seems to be reflected in the geographical range of *D. longipes*, which is distributed in both Peninsular Malaysia and Sumatra. Repeated inundations of the continental shelf could have promoted lineage diversification in *Disepalum* by fragmenting wider distributions across the Sunda Shelf and isolating parts of previously contiguous populations.

## Conclusions

The inferred vicariance event between the New World genus *Asimina* and the Southeast Asian genus *Disepalum* in the Oligocene (Fig. 1) is temporally congruent with the boreotropics hypothesis, suggesting that the intercontinental disjunction was caused by the large-scale disruption of a northern mid-latitude corridor of tropical vegetation due to climate deterioration in the late Eocene and early Oligocene. Ancestral area optimizations furthermore suggest that the crown of the *Disepalum* lineage (Fig. 2) had a wider distribution across western Malesia and continental Southeast Asia or exclusively in western Malesia. The genus subsequently diverged into two clades, representing the two subgenera. Southeast Asia as a whole is inferred to be the most likely ancestral area for subgen. *Enicosanthellum*, with a subsequent vicariance event between western Malesia and continental Asia identified in the late Miocene to Pliocene (Fig. 2). Ecological niche modelling and niche comparisons of sister lineages suggest that altitude contributes relatively little to species distributions within *Disepalum*. Seasonality of temperature and precipitation, which are the major contributors to the detrended principal components, have greatly influenced the geographical range of species. Conspicuous climate niche divergence, however, can only be identified once in *Disepalum* for the split between *D. pulchrum* and the other members of subgen. *Enicosanthellum*, whereas climate niches in subgen. *Disepalum* appear to have been relatively conserved. This indicates that climate niche differentiation was unlikely to have been a major driver of diversification in the genus, and other biotic or abiotic factors must be considered. Given that most diversification has been inferred to have occurred in western Malesia in the Pliocene and Pleistocene, it can be hypothesised that repeated sea level changes, which resulted in inundations of the Sunda Shelf in the Pliocene and Pleistocene, may have promoted allopatric speciation by fragmenting broader distributions.

## Additional file

Additional file 1: Table S1. Voucher information and GenBank accession numbers. **Table S2.** Number of true occurrences and pseudo-occurrences for each of the 18 species. **Table S3.** Mean values for the nine selected topological and bioclimatic variables based on distribution data of the 18 species sampled in the study. **Table S4.** Relative contribution of each topological and bioclimatic variable to niche models constructed by Maxent. **Table S5.** The optimized mean values for selected topological and bioclimatic variables of the internal nodes of the phylogeny. **Table S6.** Values of Schoener’s [58] *D* statistics for niche comparison between species by ENMTools. **Table S7.** Values of *I* statistics for niche comparison between species by ENMTools (XLSX 37 kb)
